# Inferior Vena Cava Resection Using Modified Veno-Venous Bypass With Renal Vein Drainage: Case Report Adapted From Liver Transplant Practice

**DOI:** 10.1097/MAT.0000000000002654

**Published:** 2026-01-21

**Authors:** Guido Fallani, Alice Assirelli, Matteo Ravaioli, Pietro Piazza, Riccardo Schiavina

**Affiliations:** From the * Department of Hepatobiliary Surgery and Transplantation, IRCCS Azienda Ospedaliero-Universitaria di Bologna, Bologna, Italy; † Department of Medical and Surgical Sciences (DIMEC), University of Bologna, Bologna, Italy; ‡ Department of Urology, IRCCS Azienda-Ospedaliero-Universitaria di Bologna, Bologna, Italy.

**Keywords:** veno-venous bypass, solitary kidney, vena cava reconstruction, kidney vascular exclusion, case report

## Abstract

We describe an innovative application of a modified three-arm veno-venous bypass (VVB) in the management of a complex case of recurrent renal cell carcinoma (RCC) invading the inferior vena cava (IVC) and left renal vein confluence. The patient, with a solitary kidney following right nephrectomy, underwent radical tumor resection and IVC reconstruction. To preserve renal function and minimize ischemic injury, we employed an extracorporeal circuit traditionally used in liver transplantation, adapting it to include an additional cannula in the left renal vein. This configuration allowed continuous renal venous drainage during IVC clamping, limiting kidney warm ischemia to only 14 minutes. The extracorporeal circuit included jugular and femoral venous drainage limbs connected to a centrifugal pump and a third limb providing direct renal outflow, effectively maintaining hemodynamic stability and renal perfusion. Postoperative recovery was uneventful, with transient minimal creatinine elevation and no acute kidney injury. This case demonstrates the versatility of extracorporeal venous bypass circuits in complex onco-vascular surgery and highlights the potential for broader applications of organ support technologies in preserving organ function during major vascular reconstruction. The proposed configuration represents a valuable adjunct in surgeries involving solitary kidneys and prolonged caval occlusion, bridging concepts from transplant and extracorporeal support domains.

## Graphical abstract

**Figure:**
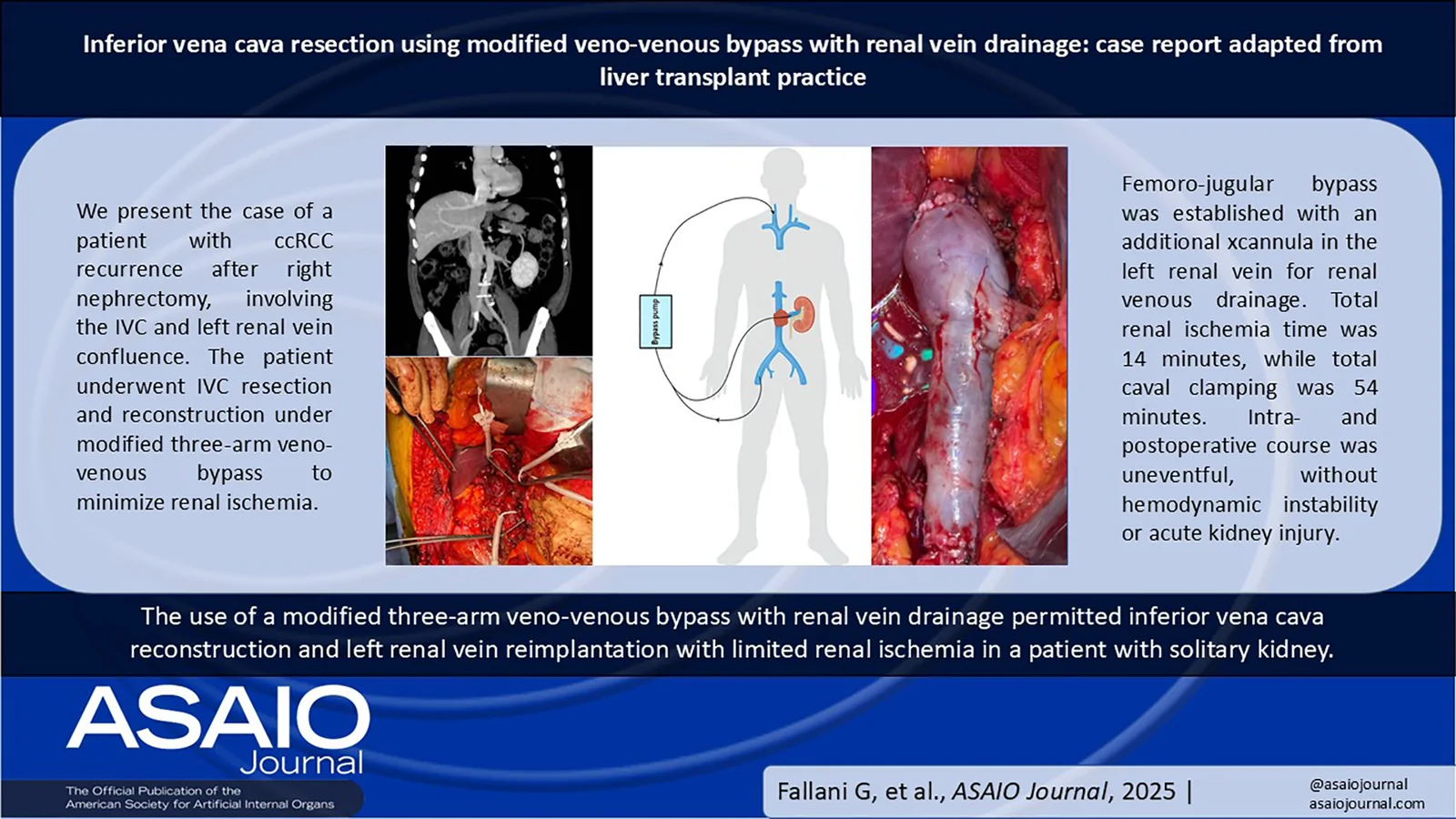

Veno-venous bypass (VVB) has been widely adopted in the practice of liver transplantation since its inception, with the aim of minimizing hemodynamic instability and splanchnic congestion caused by clamping of the inferior vena cava (IVC) and portal vein.^1^ The technique involves the placement of cannulas in the femoral and jugular veins to externally divert blood flow from the subdiaphragmatic venous system to the heart, preserving venous return and avoiding major hemodynamic perturbations. Additionally, a third cannula is typically inserted into the portal vein stump to facilitate splanchnic venous drainage, thereby reducing congestion and hypoperfusion.^2,3^ Although the introduction of surgical techniques that preserve the recipient’s retrohepatic IVC—such as side-to-side or piggyback caval anastomoses—has led to a reassessment of VVB’s role in liver transplantation, it remains a valuable adjunct and has been successfully employed in other surgeries requiring prolonged caval clamping.

We describe a case of a patient with a renal cancer recurrence following right nephrectomy, with infiltration of the IVC and the left renal vein confluence, in which a modified three-arm VVB setup was employed to allow IVC reconstruction and left renal vein reimplantation with minimal renal ischemia. The manuscript was drafted in accordance with Surgical Case Report (SCARE) guidelines.^4^ Complete written informed consent was obtained from the patient for the publication of this study and accompanying images.

## Case Presentation

**Figure 1.**
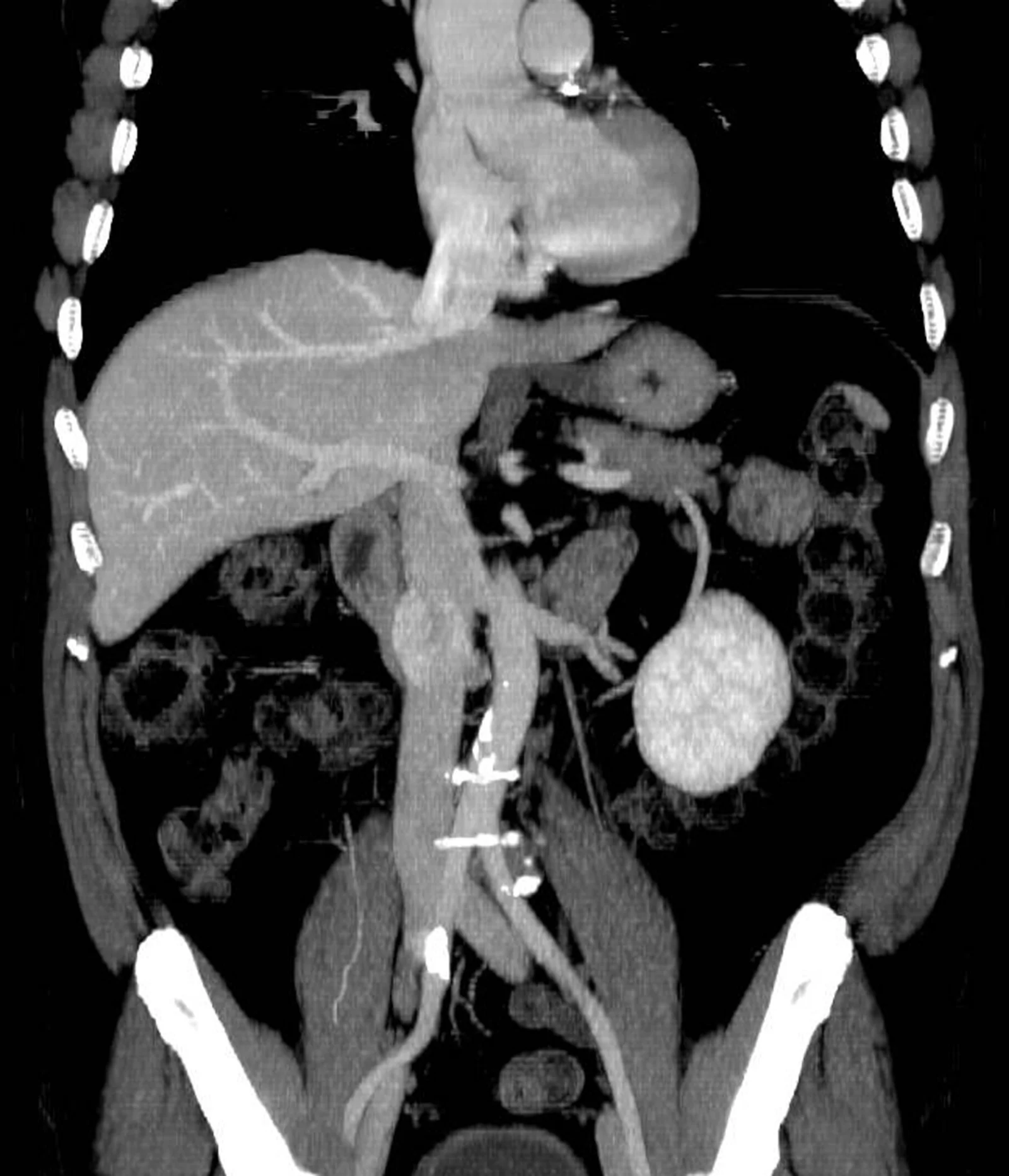
Patient’s preoperative computed tomography showing tumor recurrence involving the inferior vena cava at the level of the right renal vein stump, extending into the confluence with the left renal vein.

A 55 year old male with a history of right nephrectomy for pT3a G4 clear cell renal carcinoma (ccRCC) presented with disease recurrence 6 months postoperatively. Surveillance computed tomography (CT) revealed tumor recurrence involving the IVC at the level of the right renal vein stump, extending into the confluence with the left renal vein and causing dilation of the infrarenal vena cava, with an endoluminal filling defect due to neoplastic thrombosis. Additionally, a suspect lymph node metastasis was described in the inter-aorto-caval space (Figure 1). The patient had no relevant comorbidities.

Initially, first-line immunotherapy with nivolumab and ipilimumab was proposed, but the patient sought a second opinion at our institution. After multidisciplinary kidney tumor board review, surgical resection with IVC reconstruction and left renal vein reimplantation was recommended and accepted.

**Figure 2.**
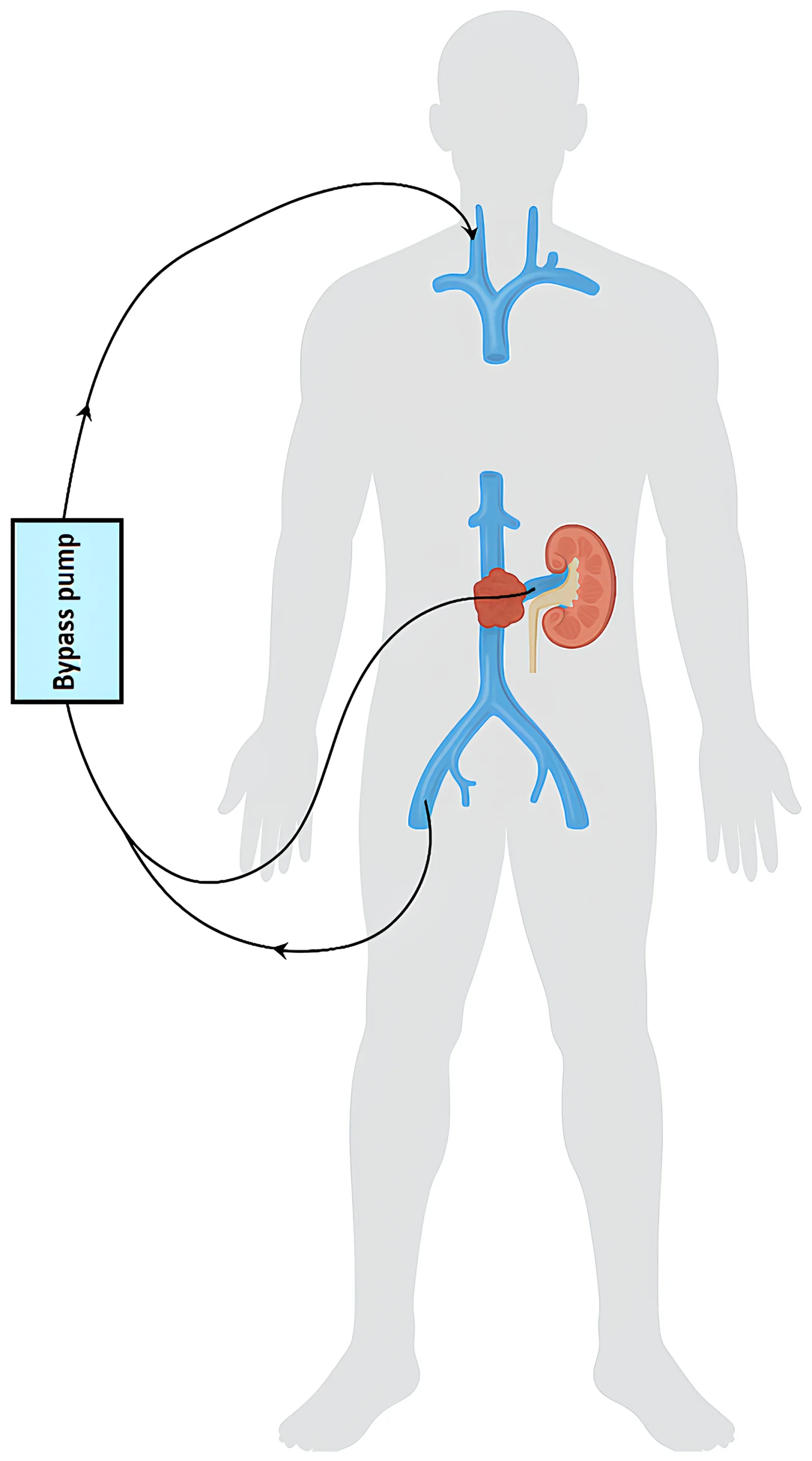
Modified three-arm veno-venous bypass setup.

Given the necessity for resection and reconstruction of the infiltrated IVC, and the anticipated prolonged caval clamping, the procedure was planned with external femoro-jugular VVB. Additionally, we decided to maintain renal vein drainage through a third bypass cannula, with the aim of minimizing ischemia of the solitary kidney and the risk of postoperative acute kidney injury (AKI). The modified three-arm VVB setup is illustrated in Figure 2.

**Figure 3.**
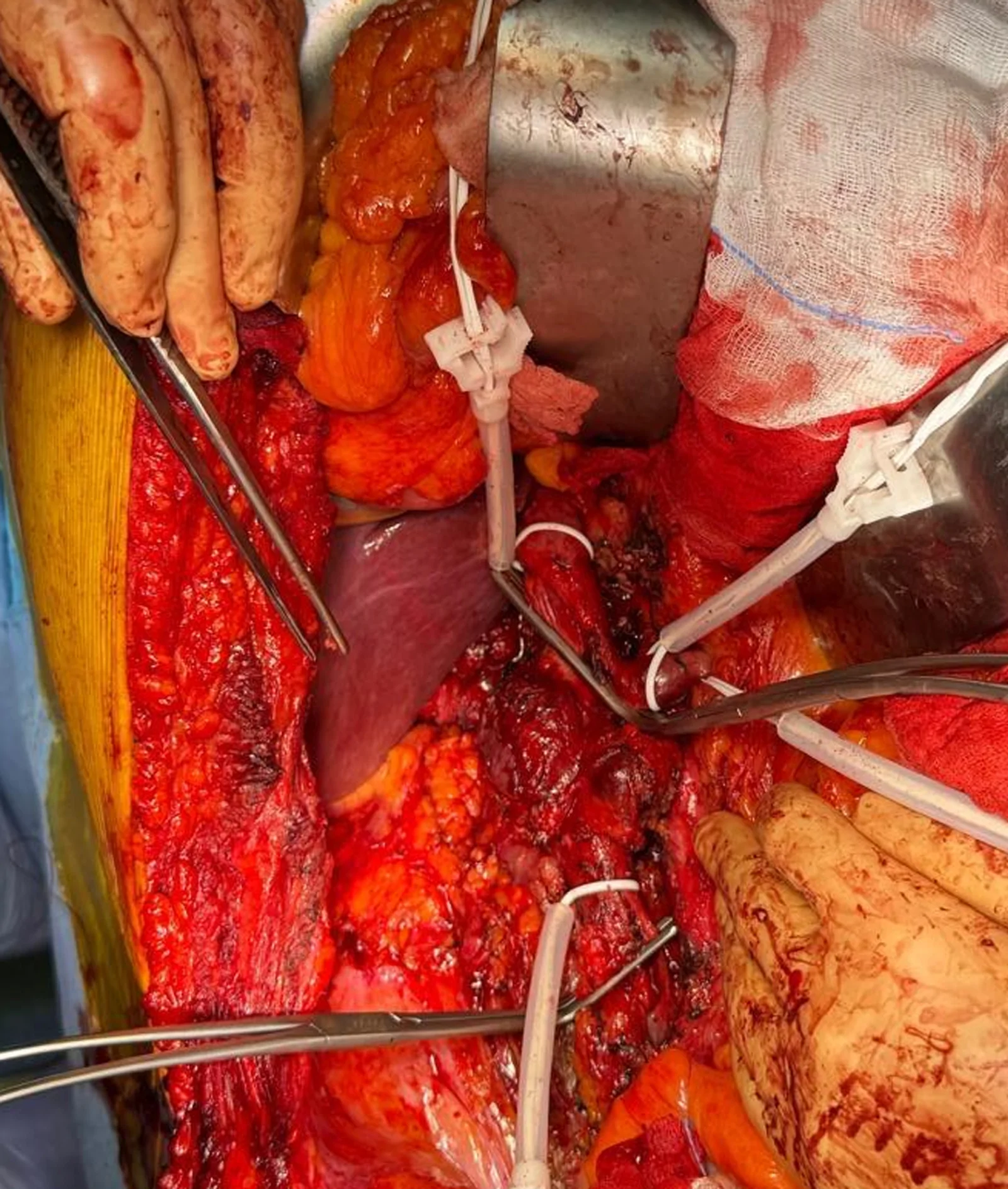
Intraoperative caval clamping setup. Rumel tourniquets are positioned on the inferior vena cava above and below the tumor, and on the left renal vein and artery.

**Figure 4.**
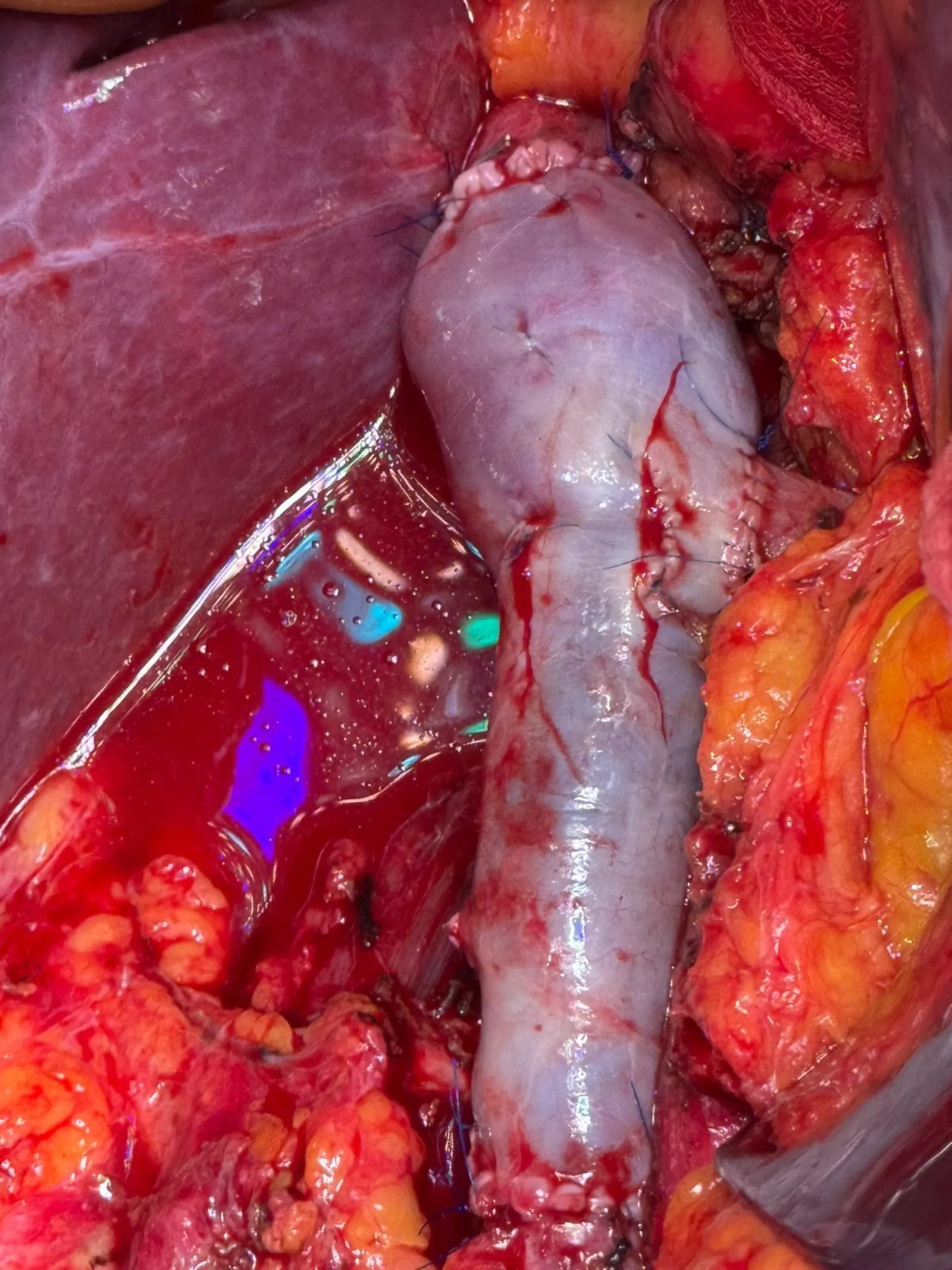
Completed allograft reconstruction of the inferior vena cava and left renal vein implantation.

The procedure started with the placement of a 16 Fr cannula in the right jugular vein and of an 18 Fr cannula in the right femoral vein, connected to the external bypass circuit with a target flow of 1.5 L/min. After the Chevron laparotomy, a Cattel–Braasch maneuver was performed. The neoplastic mass was tightly adherent to the third portion of the duodenum, necessitating tangential resection using a linear stapler, which enabled Kocher mobilization and full infrahepatic IVC exposure. The tumor was found to infiltrate a 6 cm tract of IVC at the level of the left renal vein confluence (Figure 3). Interaortocaval lymphadenectomy was performed, and the left renal artery origin was exposed and encircled for subsequent clamping. The renal artery was briefly clamped to allow left renal vein division and insertion of an 18 Fr cannula, which was connected to the VVB circuit. At this stage, the IVC was clamped above and below the tumor, and the neoplastic recurrence was excised en bloc with the IVC and the endoluminal tumor thrombus. Superior and inferior IVC margins, as well as the left renal vein margin, were clear of tumor infiltration at fresh frozen pathology evaluation. The IVC was then reconstructed with the interposition of a heterologous cryopreserved vena cava graft from a cadaveric donor, as per institutional practice.^5^ A lateral venotomy was made on the interposed graft to allow termino-lateral anastomosis of the left renal vein. The left renal artery was briefly reclamped during this phase to facilitate disconnection from the bypass and completion of the venous anastomosis (Figure 4). Veno-venous bypass was discontinued following vascular reconstruction. Patency of the graft and adequate IVC flow were confirmed via intraoperative and transesophageal ultrasound. After achieving hemostasis, the abdomen was closed, and all cannulas were removed. The total operative time was 395 minutes, with 54 minutes of IVC clamping and only 14 minutes of renal ischemia. Hemodynamic stability was maintained throughout, without the need for vasopressors.

Postoperatively, the patient was transferred to the surgical intensive care unit (ICU) and discharged to the ward on postoperative day (POD) 1. A mild increase in serum creatinine was observed (from 1.23 to 1.53 mg/dl on POD 1), with preserved urine output. Creatinine levels normalized in the following days, and the patient was discharged on POD 12 after confirming IVC and left renal vein patency via abdominal ultrasound.

Final pathology confirmed G4 ccRCC recurrence with negative margins. At 6 month follow-up, the patient is alive, in good clinical condition, and maintains preserved renal function.

## Discussion

In most cases, sudden interruption of IVC flow results in a significant drop in pulmonary artery pressure and cardiac output, with compensatory increases in heart rate and peripheral resistance mediated by vasopressin release and sympathetic activation.^6^ Although caval-sparing techniques have reduced the need for VVB in liver transplantation, many centers continue to use VVB selectively to mitigate the physiologic impact of prolonged IVC clamping.^2,3^ These hemodynamic effects are also relevant in oncologic procedures where extensive caval involvement advocates prolonged clamping and vascular reconstruction.

In the present case, left renal vascular exclusion was necessary to facilitate IVC reconstruction and venous reimplantation; however, this posed a high risk of AKI, given the solitary kidney. Ischemia–reperfusion injury, in fact, leads to endothelial injury–mediated vasoconstriction, immune activation, and tubular dysfunction and necrosis, compounding glomerular hypoperfusion through tubuloglomerular feedback. These pathophysiologic mechanisms contribute not only to AKI but also have the potential to condition renal function in the long term.^7^

Autotransplantation of the left kidney was considered but would have imposed greater surgical insult and prolonged ischemia, with higher complication risk. Instead, the adoption of VVB allowed the radical excision of a neoplastic recurrence invading the IVC with minimal impact on the patient’s hemodynamics during surgery. Moreover, the innovative adoption of a third bypass arm in the left renal vein avoided the necessity for left kidney vascular exclusion during IVC reconstruction and limited the total ischemia time for the kidney. Without renal vein cannulation, the duration of kidney vascular exclusion would have encompassed the duration of tumor excision, left renal vein reimplantation on the graft, and at least one graft anastomosis to the patient’s IVC. The modified VVB setup instead allowed us to reduce kidney ischemia to just 14 minutes, corresponding to the time employed to insert the third cannula in the renal vein (4 minutes) and later to remove the cannula and perform the anastomosis between the renal vein and the caval graft (10 minutes). This novel VVB modification contributed significantly to the patient’s uneventful recovery and preserved renal function, with only minor creatinine elevation, below the AKI threshold defined by Kidney Disease Improving Global Outcomes (KDIGO) guidelines.^8^

In conclusion, the familiarity with the VVB setup for liver transplantation allowed us to successfully adapt the technique in order to maintain renal venous return in the setting of a complex oncological surgery involving IVC reconstruction and venous reimplantation of a solitary kidney. This tailored approach enabled effective tumor resection with preserved renal function and minimal perioperative morbidity.
